# Psychometric Properties of the Toileting Behaviour Evaluation (TBE) Using Rasch Analysis

**DOI:** 10.7759/cureus.77075

**Published:** 2025-01-07

**Authors:** Yasuhiro Higashi, Toshikatsu Kaneda, Takumi Horimoto, Shuichiro Kiku, Yuta Somei, Soji Ono, Kimiaki Hirayama, Haruka Atosako, Yoshimi Yuri

**Affiliations:** 1 Faculty of Rehabilitation, Morinomiya University of Medical Sciences, Osaka, JPN; 2 Department of Rehabilitation, Osaka General Hospital of West Japan Railway Company, Osaka, JPN; 3 Department of Rehabilitation, Amagasaki Daimotsu Hospital, Amagasaki, JPN; 4 Department of Rehabilitation, Kansai Rehabilitation Hospital, Toyonaka, JPN; 5 Department of Rehabilitation, Higashiosaka Hospital, Osaka, JPN; 6 Department of Rehabilitation, Kyowakai Hospital, Suita, JPN

**Keywords:** activities of daily living (adl), rasch analysis, reliability, scale, structural validity, toileting behavior

## Abstract

This study assessed the psychometric properties of the Toileting Behaviour Evaluation (TBE), a tool designed to address the limitations of traditional assessments like the Functional Independence Measure and Barthel Index in capturing the complexities of toileting activities among wheelchair users. Conducted with 250 wheelchair users from six rehabilitation hospitals in Japan, the study used Rasch analysis to evaluate the TBE's internal validity, reliability, and unidimensionality across 22 toileting components. The analysis confirmed the TBE's fit to the Rasch model, with a raw variance explained by measures of 70.1%, a person separation index of 3.82 (reliability coefficient=0.94), and an item separation index of 5.94 (reliability coefficient=0.97), supporting its use as a valid and reliable tool. Additionally, a conversion table was developed to transform ordinal scores into interval measures, enhancing clinical applicability. The study suggests that the TBE is a psychometrically sound instrument for assessing toileting behavior in wheelchair users. However, further research is recommended to explore its effectiveness in clinical interventions and its applicability across different populations and diagnoses.

## Introduction

Toileting is a critical and frequently required task in activities of daily living (ADL). In clinical settings, it is common to encounter requests from patients in acute and rehabilitation hospitals seeking to regain independence in toileting immediately after hospitalization. The inability to perform toileting independently can significantly affect an individual's quality of life [1]. Difficulties in toileting can result in diminished functional abilities, skin infections, a heightened risk of pressure ulcers, depression, reduced social interaction, and social isolation [2-4]. Furthermore, the need for assistance with toileting not only burdens individuals but also imposes economic, mental, and physical strain on their families and society [5,6]. Additionally, the level of independence in toileting can affect the length of hospital stays [7-10]. Therefore, occupational therapists, as experts in ADL support, dedicate considerable time to evaluating and assisting with toileting activities.

Most occupational therapists use the Functional Independence Measure (FIM) or the Barthel Index (BI) for the quantitative assessment of toileting. However, these assessments cover only two items in the FIM and one item in the BI related to toileting, indicating a lack of responsiveness [11]. Toileting involves various components such as "transferring to the toilet," "managing lower garments," and "cleaning up after urination and/or defecation with toilet paper" [12,13]. Assessing individual components of ADL has been found to be effective for establishing rehabilitation goals and designing intervention plans [12]. Hence, a detailed assessment of each component of toileting activities is considered necessary for more targeted and effective interventions.

In response to this need, we developed the Toileting Behavior Evaluation (TBE), which assesses 22 components of toileting activities, such as "entering by opening the door," "transferring to the toilet," "managing lower garments," and "cleaning up after urination and/or defecation with toilet paper." The TBE evaluates these aspects of toileting activities on a 6-point ordinal scale for patients using wheelchairs. Its inter- and intra-rater reliability and concurrent validity have been verified using classical test theory [14]. The TBE was developed primarily for use in clinical settings such as acute and rehabilitation hospitals, where occupational therapists frequently encounter patients seeking to regain independence in toileting activities. Given its detailed assessment of 22 components of toileting, the TBE is particularly useful for wheelchair users in these settings. However, the applicability of the TBE can extend to other clinical environments, such as long-term care facilities or home care settings, where detailed evaluations of toileting behaviors are critical for planning personalized interventions. The flexibility of the TBE's rating scale allows clinicians to tailor its use according to the specific needs and goals of their patients. Future studies are planned to explore its utility in a wider range of clinical contexts.

The TBE was initially developed not for aggregating total scores to measure client abilities but to utilize descriptive information from ordinal rating scales for setting goals and planning interventions. However, in the field of rehabilitation, it is essential to accurately measure the outcomes of clinical interventions and objectively demonstrate their effectiveness. Therefore, rather than limiting the use of the TBE to a detailed assessment tool, we sought to employ it as a "measure" by calculating total scores that could facilitate the evaluation of intervention effects through pre- and post-intervention comparisons. Although ordinal scale scores are useful for providing detailed descriptive data, they are unsuitable for outcome measurement because they cannot be aggregated in the same manner as interval scores, which are required to produce an overall total score [15,16]. To address this issue, Rasch analysis, grounded in modern measurement theory, has been employed in rehabilitation medicine to overcome the limitations of ordinal scores. This analysis is crucial for scrutinizing the properties of existing ordinal-level instruments and affirming their measurement capabilities. Importantly, Rasch analysis offers a means of converting total scores into more meaningful measures [16].

Rasch analysis is a methodological approach that estimates individual abilities and assesses item difficulty using log odds units (logits) on a singular, continuous scale. Unidimensionality is a critical concept in this framework. Unidimensionality refers to the concept that items within a scale should collectively measure a single underlying construct. This is achieved through a hierarchical arrangement of items, ranging from those that are easy to perform to those that are more challenging, thereby reinforcing the internal validity of the scale. Before constructing a measure, the unidimensionality of items must be verified. Additionally, Rasch analysis can be applied to determine whether the hierarchical order of scale items accurately reflects sequential difficulty [16].

Unidimensionality can be explored using various analytical methods to validate the measurement potential of a scale. Goodness-of-fit analysis is a method that assesses the congruence of all individuals and items with the Rasch model. This involves examining the infit mean square (MnSq), outfit MnSq, and standardized z (Zstd) values, which indicate the alignment between the actual and expected responses. Goodness-of-fit analysis can also be used to examine the psychometric properties of rating scales. It enables the exploration of response categorizations, yielding higher-quality measures than other categorizations [17]. Another approach is the principal component analysis (PCA) of Rasch-based residuals, which is distinct from classical test theory's PCA. This method is used to evaluate whether all data align with the underlying latent measure, unlike the correlation model in classical test theory, which identifies factors on the scale [18].

Finally, Rasch analysis necessitates the exploration of person and item reliability. The person reliability index reflects the consistency in the ranking of individuals' ability logit scores if a parallel set of items were administered to the same sample. The item reliability index gauges the repeatability of item ordering along a continuum when presented to a similarly sized and behaving sample [16].

Four research questions were formulated: The first two questions address validity, the third addresses reliability, and the fourth concerns the conversion of ordinal scale scores to interval scale scores for practical application. (1) Does the TBE's rating scale exhibit appropriate psychometric properties as indicated by the ordering of category measures, the acceptable goodness of fit of the rating scale categories to the Rasch model, and the ordering of the calibration thresholds between the rating scale categories? (2) Do the items on the TBE define a single unidimensional construct using Rasch analysis? (3) Do the items on the TBE effectively separate participants into different ability levels, and do the participants, in turn, differentiate the items into various levels of difficulty? (4) How is the conversion table designed to transform raw scores from an ordinal scale to interval scale logits, utilizing rating scales and items that have been confirmed for reliability and validity through Rasch analysis?

## Materials and methods

Study design

Occupational therapists assessed the toileting behavior of the selected participants using the TBE. Additionally, data such as age, gender, diagnosis, and FIM scores were extracted from medical records.

Participants

This study recruited participants admitted to six acute and subacute rehabilitation hospitals between April 2020 and October 2023, including Kansai Rehabilitation Hospital, Osaka General Hospital of West Japan Railway Company, Kiba Hospital, and Kyowakai Hospital in Osaka, Japan, as well as Amagasaki Daimotsu Hospital and Takarazuka Rehabilitation Hospital in Hyogo, Japan. Occupational therapists at each hospital selected participants based on the inclusion and exclusion criteria established in a preceding study [14]. The inclusion criteria were as follows: eligible participants were those who could sit in a wheelchair and use it for toileting activities during training sessions or in daily living. The exclusion criteria were as follows: individuals who were physically unable to perform toileting activities (e.g., those with an indwelling urinary catheter), individuals who ambulated to the toilet, and individuals from whom consent could not be secured. To achieve stable item calibration in Rasch analysis with 99% confidence, a target sample size of 250 participants was set [19].

Ethics

This study was conducted with the approval of the Research Ethics Review Committee of Morinomiya University of Medical Sciences (approval number: 2020-004) and was conducted after obtaining consent from the participants or their families. The study adheres to the principles of the Declaration of Helsinki.

TBE

The TBE is a scale that assesses 22 components of toileting activities across six levels (6: independent, 5: modified independence, 4: supervision, 3: verbal assistance, 2: physical assistance, 1: total assistance) for participants using wheelchairs. Observations of the participants' toileting activities are used for scoring. The TBE does not employ a hierarchical structure with major or intermediate categories. Each toileting component is assessed independently to ensure that all aspects of the patient's performance are captured with precision. To evaluate the participants' maximum capability, the procedure generally starts with "supervision," progressing to "verbal assistance," "physical assistance," and "total assistance" as needed. It is a principle to not provide "physical assistance" without "verbal assistance" unless there is an emergency such as a risk of falling. "Total assistance" is scored when the participant's cooperation is absent or the task is entirely unachievable, indicating that the assistance required exceeds 75%. Non-physical support, such as pointing, is categorized under "verbal assistance" and distinguished from "supervision." Pointing, though a non-verbal action, was classified under "verbal assistance" rather than "supervision" because it serves as a direct and intentional aid often used alongside verbal instructions to enhance clarity. This classification reflects its frequent role in guiding actions more effectively than verbal cues alone. The scale's inter- and intra-rater reliability, internal consistency, and concurrent validity were established based on classical test theory [14].

Data analysis

All statistical analyses stated below were conducted using the WINSTEPS software (version 5.4.0).

Analysis of the rating scale

According to Bond et al. [16], rating scale diagnostics should be done first to confirm that the categories function as intended. This approach ensures that unidimensionality is tested after confirming the scale's proper functioning. This initial phase focused on examining the psychometric properties of the rating scale. In line with the principles set forth by Bond et al. [16], it is crucial to have at least 10 observations for every rating category to guarantee the stability of the measurements obtained. Furthermore, the average measures for each category are expected to show an increasing trend. This means that, on average, individuals scoring 5 are anticipated to show higher competence than those scoring 4, underpinning the premise that a score of 5 denotes a higher level of skill or competence than a score of 4. Additionally, the examination of fit statistics provides a method for assessing the effectiveness of the rating scale. In particular, outfit mean-square values should be under 2.0 for the rating scale to function optimally, as recommended by Bond et al. [16]. If these criteria were not met, the approach was to consolidate the categories that did not show progression and reanalyze the data.

Validity and reliability

Construct Validity

The variety of fit statistics can vary based on the test characteristics. In clinical observational assessments, an MnSq value exceeding 1.7 combined with a Zstd value greater than 2.0 indicates a misfit, suggesting inconsistencies in the test items, participant ability patterns, or the measurement structure [16]. The Rasch model provides two MnSq values, infit MnSq and outfit MnSq, with the decision to eliminate items based on infit MnSq values, which are weighted by information and hence indicate misfit more sensitively than the unweighted outfit statistic, which is influenced more by extreme scores. Concerns are typically raised more by abnormal infit statistics than by large outfit statistics [16].

Unidimensionality was assessed using PCA of Rasch model residuals, following the five-tier quality criteria established by Fisher [20] to ascertain the presence of additional factors. The criteria stipulate that the proportion of unexplained variance by the first contrast should be under 15% to qualify as "fair."

The variance accounted for by the measures in the PCA of Rasch-based residuals, previously utilized for unidimensionality evaluation, was also considered. Initially, the criterion for the variance explained by the measures was set at above 50%; however, it was subsequently modified due to shifts in the variance explanation with item-person targeting. At present, no established range of values for evaluating scale functionality exists in the literature. Consequently, this study exclusively employed criteria related to the proportion of variance unexplained by the first contrast.

In addition, to investigate the presence of differential item functioning (DIF) across gender groups, we also employed the Rasch model to identify items that function differently between male and female respondents. DIF was examined based on the difference in item difficulty estimates (logits) between gender groups. Following the criteria recommended by Linacre [21], items were considered to exhibit substantial DIF only if they demonstrated both a statistically significant difference (p<0.05) and a DIF contrast of 0.5 logits or greater. Furthermore, Linacre [22] suggests that a minimum sample size of 100 respondents per group is necessary for reliable DIF detection; therefore, the analysis in this study was limited to gender-based DIF. Additionally, we examined ceiling effects (the percentage of participants achieving the maximum possible score) and floor effects (the percentage of participants achieving the minimum possible score) to evaluate response distribution across the scale.

Reliability

The reliability indices for persons and items refer to the consistency of a person's ability logit scores and the replicability of items when administered to another sample of similar size and behavior. According to Bond et al. [16], a reliability value exceeding 0.8 is desirable, and a separation index of 2.0 indicates that the scale can differentiate at least three levels of difficulty or ability.

## Results

Participants

This study enrolled 250 participants (mean age 77.4±10.6 years), including 114 men and 136 women, with various health conditions such as cerebrovascular diseases, orthopedic disorders, and disuse syndrome. Participants' diagnoses were determined by physicians and categorized using the Major Diagnostic Category (MDC) system. Although cerebrovascular accidents (CVAs) are typically classified under the circulatory system in the MDC system, this study followed the methodology of a previous study [14] and categorized CVAs separately. This decision was made to account for the unique motor and cognitive symptoms associated with CVAs, which differ significantly from other conditions within the circulatory system category. The attributes and characteristics of the participants are summarized in Table 1.

**Table 1:** Participants' characteristics SD: standard deviation; FIM: Functional Independence Measure

Number	250
Age	
Years, mean (SD)	77.4 (10.6)
Range	27-100
Gender	
Male	114
Female	136
Diagnosis	
Cerebrovascular accident	166
Circulatory system	3
Musculoskeletal system and connective tissue	60
Nervous system	12
Respiratory system	4
Kidney and urinary tract	2
Injuries, poison, and toxic effects of drugs	1
Diseases and disorders of the digestive system	2
FIM-Motor	
Mean (SD)	44.8 (15.9)
Range	15-84
FIM-Cognition	
Mean (SD)	21.7 (7.1)
Range	7-35

Rating scale analysis

**Figure 1:**
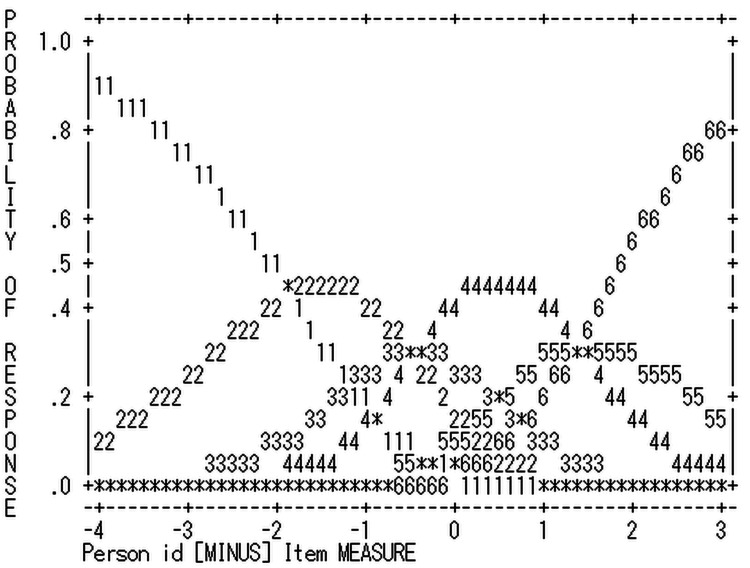
Modeled category probability curves

The six-category scale employed in the TBE was deemed acceptable based on Linacre's [20] criteria: (1) Each category had more than 10 counts, (2) the average measures increased monotonically with the category, and (3) all outfit MnSq values were below 2.0 (see Table 2). The modeled category probability curves are presented in Figure 1.

**Table 2:** Six-category rating scale analysis MnSq: mean square

Score	Frequency	%	Outfit MnSq	Calibration threshold	Category measure
1	604	11	1.11	None	-3.16
2	1005	19	1.01	-1.9	-1.47
3	785	15	0.91	-0.46	-0.43
4	1145	21	0.74	-0.45	0.5
5	605	11	0.9	1.42	1.55
6	1192	22	1.25	1.4	2.93

Validity and reliability analysis

Construct Validation

When the dataset encompassing all 22 items was analyzed, no misfit items were identified. For detailed item measurement reports, refer to Table 3. The item "Pull the lower garments up" was found to be the most difficult task, whereas "Maintain a sitting position on the toilet seat" was the easiest. The PCA results from the unidimensionality assessment of these 22 items showed that the measures accounted for 70.1% of the total variance and the first contrast explained 10.4% of the residual variance.

**Table 3:** Item measurement report SE: standard error; MnSq: mean square; Zstd: standardized z

Item	Item difficulty	SE	Infit	
			MnSq	Zstd
Pull the lower garments up	0.82	0.07	0.88	-1.33
Pull the lower garments down	0.64	0.07	0.94	-0.61
Maneuver the wheelchair to the appropriate place for transfer to the toilet seat	0.53	0.07	1.47	4.54
Close the door	0.39	0.07	1.31	3.13
Turn while standing (toilet to wheelchair)	0.35	0.07	0.61	-4.93
Turn while standing (wheelchair to toilet)	0.33	0.07	0.62	-4.85
Open the door and exit the toilet room	0.29	0.07	1.33	3.26
Open the door	0.22	0.07	1.29	2.92
Flush the toilet	0.09	0.08	1.6	5.25
Stand up from the wheelchair	0.09	0.07	0.64	-4.45
Stand up from the toilet seat	0	0.07	0.72	-3.34
Sit on the wheelchair seat	-0.05	0.07	0.78	-2.55
Lock the wheelchair brakes	-0.09	0.07	0.94	-0.69
Place feet on the footrest	-0.13	0.07	0.83	-1.87
Take the footrests up	-0.2	0.07	0.85	-1.57
Maintain a standing position (before taking off pants)	-0.2	0.07	0.84	-1.8
Maintain a standing position (before putting on pants)	-0.2	0.07	0.88	-1.28
Sit on the toilet seat	-0.21	0.07	0.89	-1.22
Unlock the wheelchair brakes	-0.35	0.07	0.82	-1.98
Clean up after urination and/or defecation with toilet paper	-0.36	0.07	1.47	4.43
Turn on the light	-0.39	0.09	1.55	3.91
Maintain a sitting position on the toilet seat	-1.56	0.08	0.78	-2.34

**Figure 2:**
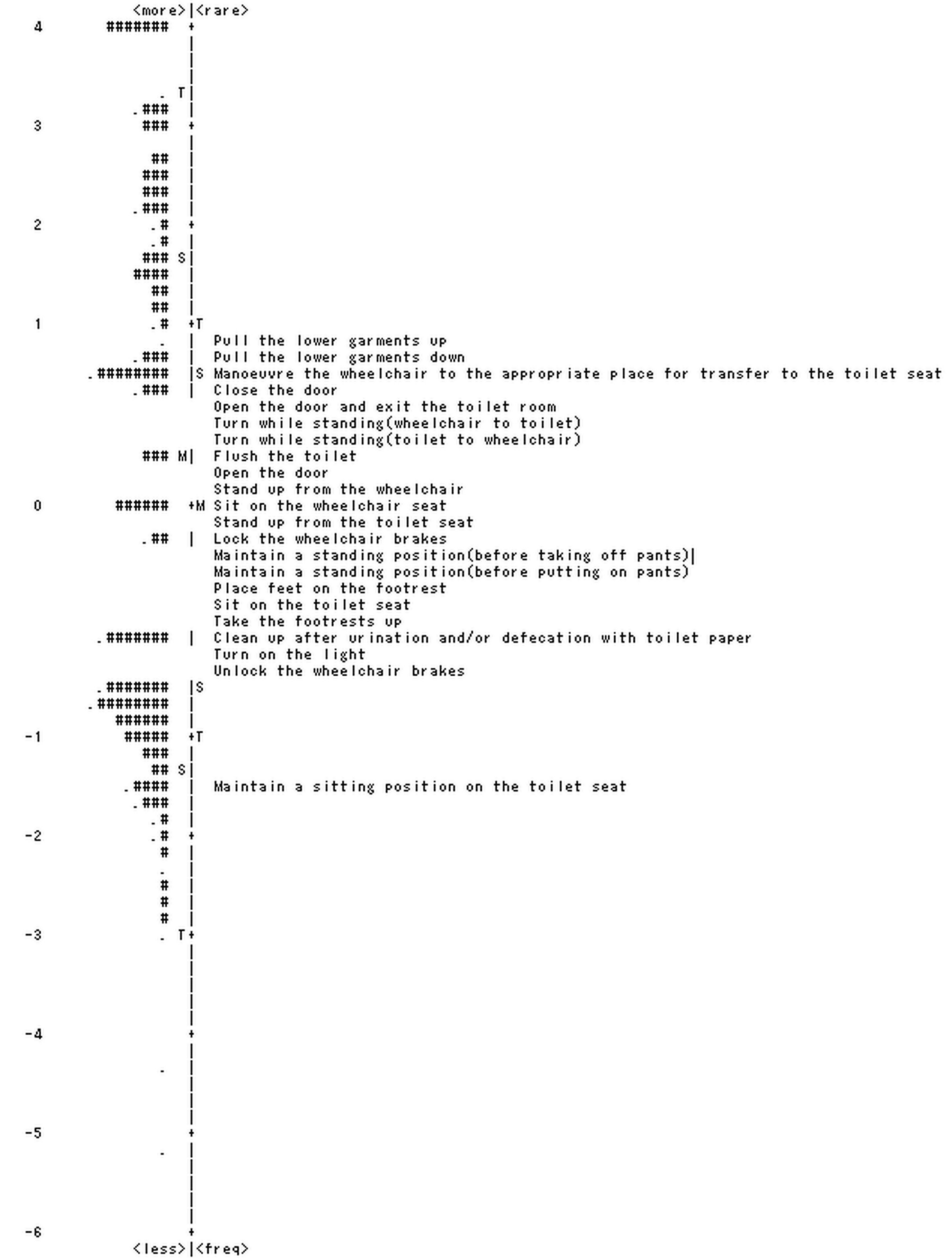
Item-person map indicating the spread of items and people along the TBE scale Each item appears in the column indicating the rating scale measure: . = 1 person and # = 2 persons TBE: Toileting Behavior Evaluation

Regarding DIF, while three items, namely, "Open the door," "Open the door and exit the toilet," and "Close the door," showed statistically significant differences, no DIF was detected, as all DIF contrasts were below the threshold of 0.5. The floor effect was not observed, and the ceiling effect was 4.8% (with 12 participants achieving the maximum score). The distribution of items and persons (item-person map) is shown in Figure 2.

Reliability

The reliability analysis indicated a person separation index of 3.82, suggesting that the TBE could categorize participants into at least four distinct competence levels in toileting behavior, with a separation reliability coefficient of 0.94. The item separation index was 5.94, with a separation reliability coefficient of 0.97.

Conversion table

The conversion table for the six-category, 22-item scale, which meets the criteria for reliability and validity, is presented in Table 4. The total raw scores range from 22 to 132 points, and when converted to logits, the range is from -6.33 logits to 5.81 logits.

**Table 4:** Conversion table SE: standard error

Score	Measure	SE	Score	Measure	SE	Score	Measure	SE
22	-6.33E	1.84	59	-0.75	0.21	96	0.88	0.22
23	-5.1	1.02	60	-0.7	0.21	97	0.93	0.22
24	-4.38	0.73	61	-0.66	0.21	98	0.98	0.22
25	-3.94	0.6	62	-0.61	0.21	99	1.03	0.22
26	-3.62	0.53	63	-0.57	0.21	100	1.08	0.22
27	-3.37	0.48	64	-0.52	0.21	101	1.13	0.22
28	-3.17	0.44	65	-0.48	0.21	102	1.18	0.22
29	-2.99	0.41	66	-0.44	0.21	103	1.23	0.23
30	-2.83	0.39	67	-0.39	0.21	104	1.28	0.23
31	-2.69	0.37	68	-0.35	0.21	105	1.33	0.23
32	-2.56	0.35	69	-0.31	0.21	106	1.38	0.23
33	-2.44	0.34	70	-0.27	0.21	107	1.44	0.23
34	-2.34	0.32	71	-0.22	0.21	108	1.49	0.23
35	-2.23	0.31	72	-0.18	0.21	109	1.54	0.24
36	-2.14	0.3	73	-0.14	0.21	110	1.6	0.24
37	-2.05	0.29	74	-0.1	0.21	111	1.66	0.24
38	-1.97	0.29	75	-0.05	0.21	112	1.72	0.25
39	-1.89	0.28	76	-0.01	0.21	113	1.78	0.25
40	-1.81	0.27	77	0.03	0.21	114	1.84	0.25
41	-1.74	0.27	78	0.07	0.21	115	1.91	0.26
42	-1.67	0.26	79	0.12	0.21	116	1.98	0.26
43	-1.6	0.26	80	0.16	0.21	117	2.05	0.27
44	-1.53	0.25	81	0.2	0.21	118	2.12	0.28
45	-1.47	0.25	82	0.25	0.21	119	2.2	0.28
46	-1.41	0.24	83	0.29	0.21	120	2.28	0.29
47	-1.35	0.24	84	0.34	0.21	121	2.37	0.3
48	-1.29	0.24	85	0.38	0.21	122	2.46	0.31
49	-1.24	0.23	86	0.43	0.21	123	2.57	0.33
50	-1.19	0.23	87	0.47	0.21	124	2.68	0.35
51	-1.13	0.23	88	0.51	0.21	125	2.81	0.37
52	-1.08	0.23	89	0.56	0.21	126	2.95	0.4
53	-1.03	0.22	90	0.61	0.21	127	3.12	0.43
54	-0.98	0.22	91	0.65	0.21	128	3.33	0.48
55	-0.93	0.22	92	0.7	0.21	129	3.6	0.56
56	-0.88	0.22	93	0.74	0.22	130	3.98	0.69
57	-0.84	0.22	94	0.79	0.22	131	4.64	0.98
58	-0.79	0.21	95	0.84	0.22	132	5.81E	1.81

## Discussion

The objective of this investigation was to assess the psychometric properties of the TBE using Rasch analysis. These findings offer preliminary evidence of the internal validity and reliability of the scale. Specifically, the TBE exhibited an overall acceptable fit to the Rasch rating scale model, affirming its internal validity.

Although all criteria were met, the examination of unidimensionality for internal validity revealed that two items ("Flush the toilet" and "Turn on the light") had infit MnSq values exceeding 1.5. This study collected data from six acute care and rehabilitation hospitals. Owing to the standardized facility criteria for hospitals in Japan, toilet fixtures, such as the height of toilets and placement of handrails, are consistent. However, finer details, such as the location of light switches and the mechanism for flushing, vary between hospitals, possibly affecting the difficulty level of these items and resulting in MnSq values of over 1.5. Nonetheless, the range of criteria and the fact that the percentage of the Rasch factor explained by the measures exceeded 60% supports the unidimensionality of the TBE with its 22 items.

For the distribution of responses across the scale, and based on the criteria outlined by Fisher [20], the floor effect was deemed excellent, while the ceiling effect was classified as fair. These results align with findings from other studies on ADL scales [23]. It is generally understood that ADL assessments may demonstrate some level of ceiling effect, as certain high-performing participants can reach the maximum score [24]. Nevertheless, as long as the TBE is used with participants who cannot perform toileting tasks independently, the ceiling effect is unlikely to pose significant clinical challenges. Consequently, it is feasible to calculate the total score for 22 items across a six-category scale using the TBE.

In light of these findings, we propose renaming the "Toileting Behavior Evaluation (TBE)" to the "Toileting Behavior Scale (ToBS)." Through Rasch analysis, the ToBS has proven to be a valid and reliable quantitative instrument for assessing toileting behavior, offering precise measurements that can significantly inform both clinical interventions and research analyses.

Prior to this study, no study had been conducted on the unidimensionality of the items of toileting behavior. Previous studies, such as those by Kawanabe et al. [13] and Kitamura et al. [12], reported the difficulty levels of toileting behavior tasks among stroke patients (see Table 5). Both this study and Kawanabe et al.'s study [13] found that manipulating lower garments was the most challenging task. The high difficulty level of lower garment manipulation is attributed to the dual task of maintaining a standing posture while performing the task. Kitamura et al. [12] reported that the most challenging task for stroke patients upon admission to a rehabilitation hospital was locking wheelchair brakes, which was likely related to their initial experience with wheelchair use upon hospital admission.

**Table 5:** Comparison of the item difficulty among the preceding studies TBE: Toileting Behaviour Evaluation; TTAF: Toileting Tasks Assessment Form

TBE	TTAF	Kawanabe et al.'s study
Pull the lower garments up	Lock the wheelchair brakes	Wearing pants
Pull the lower garments down	Turn while standing	Standing up from the toilet
Maneuver the wheelchair to the appropriate place for transfer to the toilet seat	Pull the lower garments down	Changing direction to wheelchair
Close the door	Turn while standing	Changing direction to the toilet
Turn while standing (toilet to wheelchair)	Pull the lower garments up and adjust them	Taking off pants
Turn while standing (wheelchair to toilet)	Maintain a standing position	Sitting on the wheelchair
Open the door and exit the toilet room	Maintain a standing position	Standing up from the toilet
Open the door	Maneuver the wheelchair toward the appropriate place for transfer to the toilet seat	Sitting on the toilet bowl
Stand up from the wheelchair	Put the foot on the footrest	Wiping the buttocks
Flush the toilet	Dispose incontinence pad/sanitary items	Cutting the toilet paper
Stand up from the toilet seat	Take the foot off the footrest and place it on the ground	
Sit on the wheelchair seat	Unlock the wheelchair brakes	
Lock the wheelchair brakes	Stand up from the toilet seat	
Place feet on the footrest	Stand up from the wheelchair	
Maintain a standing position (before taking off pants)	Sit on the wheelchair seat	
Maintain a standing position (before putting on pants)	Exit the toilet room	
Take the footrests up	Clean up after urination and/or defecation	
Sit on the toilet seat	Sit on the toilet seat	
Unlock the wheelchair brakes	Open and close the door	
Clean up after urination and/or defecation with toilet paper	Press the nurse call button	
Turn on the light	Press the nurse call button	
Maintain a sitting position on the toilet seat	Open and close the door	
	Maintain a sitting position on the toilet seat	
	Flush the toilet	

Additionally, the differences in task difficulty observed between this study and previous research may be related to the conditions of the participants. In previous studies, participants only had stroke, whereas this study included participants with conditions other than stroke, such as musculoskeletal disorders or disuse syndrome. Stroke patients often experience motor paralysis, making tasks such as transferring and standing up relatively more difficult. Consequently, in previous studies, the difficulty levels of tasks related to mobility may have been higher.

Limitations

This study has several limitations. A notable limitation stems from the study's participant pool, which was drawn exclusively from six acute and subacute rehabilitation hospitals in Japan. This raises questions about the generalizability of the findings to diverse populations, especially potential cultural and infrastructural disparities in hospital settings. The specificity of this study suggests the need for cross-cultural validation studies to ascertain the effectiveness and adaptability of the ToBS in various settings, with differences in toilet facilities.

Expanding the scope of ToBS to encompass a wider array of disabilities and conditions has emerged as a critical future direction. The current focus on wheelchair users offers valuable insights but also delineates the applicability of the assessment. Additionally, the study's DIF analysis was limited by sample size, as Linacre [22] recommends at least 100 respondents per group for reliable DIF detection. This constraint restricted our analysis to gender-based DIF only. Future research should aim to increase the sample size to allow for more comprehensive DIF assessments across various diagnostic categories and other subgroups, which would enable a fuller understanding of DIF in the ToBS.

Future research should explore the reliability and validity of the ToBS among individuals utilizing various mobility aids and populations with diverse rehabilitation needs. This expansion is pivotal to ensure the comprehensive applicability of the tool and facilitate its adoption in a broad range of clinical settings and rehabilitation contexts.

## Conclusions

This study assessed the psychometric properties of the ToBS using Rasch analysis to confirm its internal validity and reliability. The results indicated that the ToBS generally fits the Rasch model, thereby supporting the scale's overall unidimensionality. This finding underscores the feasibility of calculating total scores for toileting behavior using the ToBS.

As the first study to focus on the unidimensionality of items related to toileting behavior, this research facilitated pre- and post-intervention comparisons, as well as between-group comparisons in intervention studies. Additionally, it included the development of a conversion table to quantitatively evaluate the effectiveness of interventions.

However, the study has some limitations. The participants were exclusively drawn from a limited number of facilities in Japan, which cautions against generalizing the findings. Future research should aim to extend the applicability of the ToBS to individuals with a variety of disabilities and conditions to further examine its reliability and validity. This expansion is essential for ensuring the comprehensive applicability of the ToBS and promoting its use across a broad range of clinical settings.
